# Cardiovascular and Thermal Responses to Cold Exposure During Exercise in Iron-Deficient Anemic Individuals

**DOI:** 10.3390/jfmk10030362

**Published:** 2025-09-22

**Authors:** Panagiotis Miliotis, Spyridoula Ntalapera, Panagiotis Lakeas, Argyris Toubekis, Nickos Geladas, Maria Koskolou

**Affiliations:** 1 Division of Sport Medicine and Biology of Exercise, School of Physical Education and Sport Science, National and Kapodistrian University of Athens, 17237 Athens, Greece; pmiliotis@phed.uoa.gr (P.M.); sntalapera@phed.uoa.gr (S.N.); panos.lakeas@otago.ac.nz (P.L.); ngeladas@phed.uoa.gr (N.G.); 2 Division of Sciences, School of Physical Education, Sport and Exercise Sciences, University of Otago, Dunedin 9054, New Zealand; 3 Division of Aquatic Sports, School of Physical Education and Sport Science, National and Kapodistrian University of Athens, 17237 Athens, Greece; atoubekis@phed.uoa.gr

**Keywords:** cardiovascular strain, cold intolerance, heat production, vasoconstriction, sympathetic response, temperature regulation, thermal sensation

## Abstract

**Background**: Temperature regulation is impaired in iron-deficient anemic humans and rats at rest during cold exposure. However, there is a paucity of data regarding the interplay of cold exposure, anemia, and exercise on thermal and cardiovascular responses. Therefore, we aimed to explore thermal and cardiovascular responses of individuals with chronic mild iron-deficiency anemia during exercise in the cold compared to controls. **Methods**: Nine anemic (5 F, 4 M) and nine control (5 F, 4 M) individuals, matched for body fat, size, and mass but different by design in hematological parameters and physical fitness, participated in the study. The participants cycled in cold 11 °C with 40% relative humidity (RH) and neutral (22 °C, 40% RH) conditions at an intensity ~10% below the respiratory threshold until 1 °C increase in rectal temperature (T_re_) or 1 h of exercise, whichever occurred first. **Results**: In the cold, the anemic individuals showed a lower rate of T_re_ rise (*p* = 0.047) and lower mean skin temperature (T_sk_) (*p* = 0.03) compared to controls, while only controls increased heat production compared to the neutral condition (*p* = 0.035). Moreover, the anemic group exhibited an exaggerated blood pressure response in the cold compared to the neutral environment (*p* < 0.05), due to heightened total peripheral resistance (*p* < 0.05) and vasomotor response (*p* < 0.001). **Conclusions**: In summary, chronic mild iron-deficiency anemia impaired temperature regulation as judged from the lower rate of T_re_ rise and an inability to activate further the metabolism at cold. Concomitantly, the anemic participants demonstrated increased cardiovascular strain. This is notable because anemia and these environmental conditions are encountered in the workplace, recreational activities, and athletic endeavors. These findings may inform safety guidelines for athletes, workers, and patients exposed to cold environments.

## 1. Introduction

Iron-deficiency anemia affects cardiovascular response and induces metabolic and hormonal disturbances, both of which are essential for temperature regulation [1,2,3]. Early studies in resting humans and animals with iron-deficiency anemia have shown impaired thermoregulation and altered metabolism during cold air or water exposure [4,5,6,7]. For instance, anemic individuals experienced greater body heat losses from impaired metabolism caused by enzymatic depletion of iron, reflected in oxygen uptake responses and lower rectal temperature [2,5]. These disturbances, at rest, are reversed when iron-deficiency anemia is treated, highlighting the importance of adequate iron levels for maintaining proper thermoregulation and overall metabolic function [4].

Exercise and a cold environment could independently tax cardiovascular responses [8]. It has been documented that during exercise in the cold, the cardiovascular strain is amplified, causing altered sympathetic function [9]. Cold exposure is characterized by a sympathetic nervous system excitation that causes cutaneous vasoconstriction [10] and elevated muscle sympathetic nerve activity [11], leading to increases in arterial blood pressure, and, thus, heightening the risk of cardiovascular events more pronouncedly in individuals with underlying cardiovascular diseases [10,12,13,14,15]. However, despite elevated catecholamine levels in anemic individuals at cold [16,17,18] and their disadvantageous cardiovascular system in terms of oxygen delivery, there is a paucity of studies investigating the integrated responses of thermoregulatory and cardiovascular responses.

Iron-deficiency anemia is most prevalent in well-trained premenopausal women, athletes, military personnel, and pregnant women [19,20,21]. It also coexists in various diseases such as heart failure, hypertension, kidney failure, and cancer [22,23,24,25]. Therefore, it is of great importance to examine the physiological and thermal behavioral responses of individuals with chronic iron deficiency during exercise in moderately cold environments, as these conditions are common in work, sports, and recreational activities.

This study aims to investigate how individuals with mild iron-deficiency anemia regulate body temperature and respond to cardiovascular stress during exercise in moderate cold (11–12 °C) conditions. Understanding these responses is critical for improving the safety and performance of vulnerable populations under such environmental conditions.

We hypothesized that anemic participants compared to controls would demonstrate compromised heat production during the cycling exercise in the cold, resulting in a slower rise in core temperature accompanied by exaggerated vasoconstrictor and cardiovascular responses.

## 2. Materials and Methods

### 2.1. Participants

Eighteen physically active young adult participants, nine (5 females, 4 males) controls with normal hemoglobin concentration ([Hb]), hematocrit (Hct) serum iron (Fe), and ferritin and nine (5 females, 4 males) with chronic mild iron-deficiency anemia (inclusion hematological criteria were as follows: 10 < [Hb] < 12 g/dL, 34 < Hct < 37%, and ferritin < 15 µg/L) [26] volunteered for the study (Table 1). An a priori power analysis (G Power 3.1.9.7. software) indicated that a minimum of sixteen participants (8 per group) was required to achieve a statistical power greater than 0.85, effect size η_p_^2^ > 0.06 (medium), and α value ≤ 0.05 in a mixed 3-way ANOVA design (group × condition × time).

Both groups had no previous history of cardiovascular and pulmonary diseases and were free of musculoskeletal injuries and thyroid diseases. Before visiting the lab, the participants were instructed to avoid heavy meals (4 h), intense exercise (24 h), and to not consume alcohol and caffeinated beverages (12 h). Anemic participants were not taking any iron supplementation throughout the study period. All female participants had regular menstrual cycles and were not using contraception pills. In addition, to avoid the confounding effects of female hormones on core temperature and physiological responses, all female participants exercised during the follicular phase of the menstrual cycle [27], which was assessed with urine sticks. The experimental procedures had the approval of the local Ethical Committee (1157/11-12-2019). All possible risks and benefits of the procedures were thoroughly explained to the participants, and their written consent was obtained. The two groups were well matched for age, height, body mass, body surface area, and %fat. However, they were different by design for fitness level using the prediction equation of differences in VO_2max_ with corresponding differences in hemoglobin concentration as reported by Calbet et al. [28]. More specifically, initially, fitness and hematological parameters of the anemic participants were measured, and thereafter they were matched with control participants based on [Hb]-corrected VO_2max_ values [29].

### 2.2. Study Design

Prior to the main experiments, the participants visited our lab on two occasions, including a baseline blood status assessment, a seven-site body fat calculation [30], and a familiarization with experimental procedures and the equipment/instrumentation. Thereafter, on two different days, with at least a 48 h interval, the participants performed VO_2max_ tests on a cycle ergometer (Lode, Groningen, The Netherlands), one in a cold (11 °C and RH: 40%) and one in a neutral (22 °C and RH: 40%) environment, following a randomized order in a similar way for both groups. The load of incremental exercise was initially set at 20 W for all participants and then increased by 20 W every minute for females and by 30 W for males until volitional exhaustion. The highest 20 s values were averaged to determine VO_2max_.

During experimental protocols, 1 h before the experiment, participants arrived at the lab and ingested 5 mL/BM of water. They then emptied their bladder, and their body mass was measured (Bilance Salus, Milan, Italy) while wearing only shorts (males) or shorts and a fitted top (females). Afterward, their capillary blood Hct% and [Hb] were determined. Then, they remained seated for 5 min while baseline rectal and skin temperatures and blood pressure were recorded, serving as idle values. If rectal temperature deviated by ±0.2 °C from the value recorded in the first visit, the experiment was postponed for another day. The participants remained seated for another 20 min in either a neutral or cold environment in a randomized and counterbalanced order. Then, they performed the prolonged cycling exercise protocol in the corresponding environmental condition (neutral: 22 °C, 40% RH, and cold: 11 °C, 40% RH) and (ANN: anemia + neutral, ANC: anemia + cold, and CONN: control + neutral, and CONC: control + cold) at an intensity 10% below the second ventilatory threshold (VT2) until either their rectal temperature (T_re_) increased by 1 °C or until 1 h had passed, whichever occurred first. Participants were not allowed to drink water throughout the experiment (including the resting period and exercise) and wore the same clothing across all conditions: shorts, socks, a T-shirt, and shoes. Each exercise session was separated by an interval of at least 48 h between measurements. Also, the hydration status was assessed in every visit using Hct% values and body mass [31].

### 2.3. Measurements

Core body temperature was recorded with a probe (MSR 145, MSR Electronics GmbH, Henggart, Switzerland) at the rectum, 10–12 cm past the anal sphincter, sampled and stored at 30 s intervals by an MSR data logger (Modular Signal Recorder, MSR Electronics GmbH, Henggart, Switzerland). Skin temperature was measured at four sites (forearm, fingertip, calf, and chest) using thermocouples (MSR 147WD, MSR Electronics GmbH, Henggart, Switzerland) attached to the skin with a single piece of adhesive tape, and a weighted mean skin temperature (T_skin_) was calculated [32,33]. The forearm-fingertip temperature gradient (T_f-f_) was used as an index of skin vasomotor tone, which is reproducible during exercise [34].

The local sweat rate secreted in the forearm was measured with a ventilated capsule continuously. The temperature and humidity of air entering and exiting the capsule were measured with thermocouples (TSD 202A, BIOPAC Systems Inc., Austin, TX, USA) and capacitance hygrometers (Delta), respectively, and sweat rate (SwR) was estimated from the difference between the temperature and the humidity of inflowing and outflowing air. Temperature and humidity sensors were appropriately calibrated before their use. Also, thermal sensation was assessed with a scaled questionnaire (1, cold; 3, cool; 5, slightly cool; 7, neutral; 9, slightly warm; 11, warm; 13, hot) (ASHRAE, 1966, modified [35]). Gas exchange and ventilatory variables (VO_2_, VCO_2_, RER, and VE) were recorded continuously throughout experiments breath by breath via open-circuit spirometry (Ultima CPX, MedGraphics, Austin, TX, USA). Before each test, the gas analyzers for O_2_ and CO_2_ and the pneumotachograph were calibrated with two different gas mixtures: (a) 12% O_2_ and 5% CO_2_ balanced in N_2_ and (b) 21% O_2_ and 0.01% CO_2_ balanced in N_2_ and a 3 L syringe (Ultima CPX, MedGraphics, Austin, TX, USA), respectively. Metabolic energy expenditure was calculated as follows:

$${M(W)=\dot{V}O}_{2}\frac{\left(\frac{RER-0.7}{0.3}\right)e_{c}+\left(\frac{1-RER}{0.3}\right)e_{f}}{60}$$

where “RER” is respiratory exchange ratio (VCO_2_/VO_2_), “e_c_” is the caloric equivalent of a liter of oxygen when carbohydrates are oxidized (21.1 kJ), and “e_f_” is the caloric equivalent of a liter of oxygen when fat is oxidized (19.6 kJ).

Systolic blood pressure and diastolic blood pressure were continuously recorded beat by beat noninvasively via a photoplethysmograph with the cuff attached on the middle finger of the left hand (Finometer 2003, FMS, Arnhem, The Netherlands). Stroke volume (SV) was estimated via the Modelflow method [36], while mean arterial pressure (MAP), cardiac output (CO), and total peripheral resistance (TPR) were calculated by BeatScope software (version 1.a.). The method of photoplethysmography has been shown to have very high validity correlation (0.93–0.99) with invasive methods such as arterial line placement during dynamic exercise [37]. To minimize measurement noise, the left hand was carefully and comfortably immobilized in a stable structure so as not to constrain maximal effort during exercise. The heart rate (HR) was monitored with a standard lead II electrocardiogram (ECG 100C, BIOPAC Inc., Goleta, CA, USA). The Hct% values were determined using the Hct scale (Haematocrit Reader, Hawksley Inc., Lancing, UK) after a 5 min centrifugation of 75 μL capillary blood at 11,500 rpm (Micro Haematocrit Mk5 Centrifuge, Hawksley, Lancing, UK) with a resolution of 1% units. The [Hb] values were determined by a portable photometer (Diaspect Tm) using 10 μL of capillary blood.

### 2.4. Statistical Analysis

Normality of data was assessed using the Shapiro–Wilk test. Student’s paired two-tailed *t*-test was employed for comparing participants’ characteristics. ANOVA for two factors (group x condition) was used to analyze resting values of rectal and skin temperatures, as well as rate of rectal temperature rise, heat production values, and initiation of sweating. Three-way ANOVA was conducted to analyze cardiovascular and thermoregulatory responses with [Hb] as a between-subject factor and two within-subject factors (condition and time). The cardiovascular and thermal responses presented in the figures were analyzed for the common 20 min period of exercise since there was time variation to achieve the targeted T_re_ of 38 °C. The Tukey post hoc test was used to assess the significance of parametric analysis. For significant time-related interactions, a separate two-way ANOVA was conducted at each time point of interest. Moreover, partial eta squared as measures of effect size was used, where η_p_^2^ = 0.01 is considered small, 0.06 moderate, and >0.14 as large. A violation of normality was found for thermal sensation; thus, initially a Friedman test was conducted to discriminate overall group effects (anemia vs. control). Thereafter, a Wilcoxon signed–rank test was used to detect differences between conditions within the same group and a Mann–Whitney test for independent groups at each time point. Data is presented as mean ± sd. The significance level was set at *p* ≤ 0.05.

## 3. Results

### 3.1. Sample Characteristics

The two groups differed by design in blood variables (*p* < 0.001) and were matched for anthropometric characteristics (*p* > 0.05) (Table 1). [Hb] was 21% lower in the anemic group (*p* < 0.001), and VO_2max_ was 12.5% lower, but without reaching a significant difference (*p* = 0.06) (Table 1). No group differences for MAP were detected (*p* = 0.98) before the thermal provocation for resting data (ANN: 94 ± 9; ANC: 96 ± 7 vs. CONN: 94 ± 6, CONC: 95 ± 6). Also, there were no differences between groups for baseline values regarding T_sk_ and T_re_ in neutral and cold conditions, respectively.

### 3.2. Cardiovascular Responses

**Figure 1:**
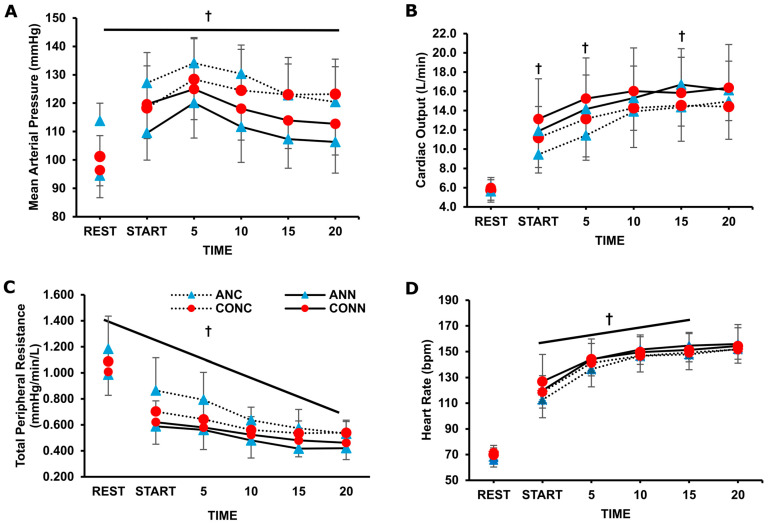
(**A**) Mean arterial pressure, (**B**) cardiac output, (**C**) total peripheral resistance, and (**D**) heart rate during rest and 20 min of exercise in anemic participants (n = 9) at neutral (ANN) and cold (ANC) and for controls (n = 9) in neutral (CONN) and cold (CONC) environmental conditions, respectively. †: significant difference between ANC and ANN (*p* < 0.05). Data are presented as mean ± sd.

There was a significant group x environment interaction for MAP (*p* = 0.031, η_p_^2^ = 0.26) and for TPR (*p* = 0.043, η_p_^2^ = 0.23), and post hoc analysis indicated that they were significantly higher in the cold condition compared to neutral, but only in the anemic group did the values reach significance, both at rest and during exercise (*p* < 0.001) (Figure 1).

For CO responses, an environment x time interaction was revealed (*p* = 0.004, η_p_^2^ = 0.19), and further analysis indicated that only in the anemic group did the CO differ at specific time points between cold and neutral conditions during exercise (Figure 1B). Overall stroke volume increased from rest to exercise (*p* = 0.001, η_p_^2^ = 0.19), and there was no difference in the cold (97 ± 26 mL) compared to the neutral (103 ± 26 mL) condition during exercise (*p* = 0.10). Overall heart rate increased during exercise in both groups and conditions (*p* < 0.001, η_p_^2^ = 0.98), and it was lower from the onset until the 15th min of exercise in ANC compared to ANN (*p* = 0.01, η_p_^2^ = 0.40). Diastolic blood pressure was higher in ANC (93 ± 10 mmHg) compared to ANN (82 ± 10 mmHg) (*p* = 0.022, η_p_^2^ = 0.75), while it was elevated in CONC compared to CONN only at the 15th and 20th min of exercise (*p* = 0.005). Systolic blood pressure was elevated only in ANC (175 ± 21 mmHg) compared to ANN (155 ± 21) (*p* = 0.0004, η_p_^2^ = 0.37), with no difference between CONC (166 ± 23 mmHg) and CONN (162 ± 23 mmHg) (*p* = 0.64).

### 3.3. Thermoregulatory Responses

The rate of rectal temperature rise was lower in ANC compared to CONC (*p* = 0.047, η_p_^2^ = 0.22) and ANN (*p* = 0.003, η_p_^2^ = 0.33) (Table 2). Accordingly, the time required to increase Tre by +1 °C was significantly longer in ANC compared to ANN and CONC (*p* = 0.001, η_p_^2^ = 0.52) and CONC (*p* = 0.021, η_p_^2^ = 0.26) (Table 2).

**Figure 2:**
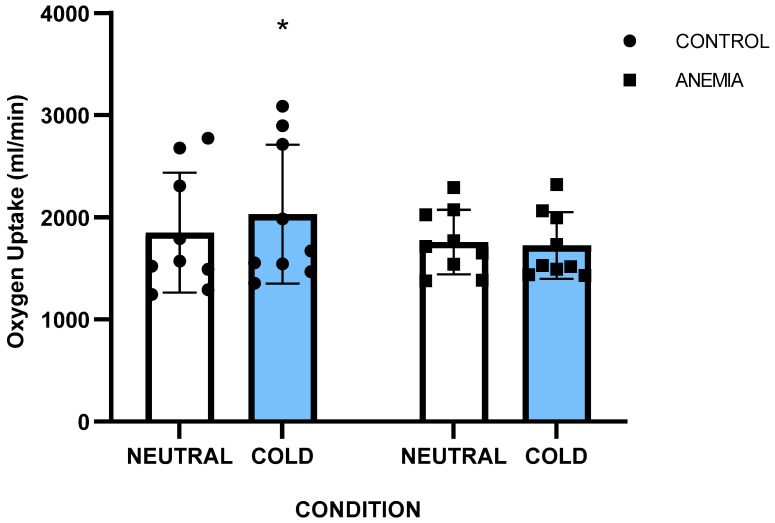
Oxygen uptake responses during exercise for anemic (n = 9) and control (n = 9) participants, in neutral and cold environmental conditions. *: Significant difference between environments (*p* = 0.036). Data are presented as mean ± sd.

A significant group x condition interaction was noted for oxygen uptake (*p* = 0.016, η_p_^2^ = 0.31), and post hoc analysis revealed that VO_2_ was higher only in the control group during exercise in the cold (CONC: 1852 ± 626 mL/min) compared to thermoneutral (CONN: 2031 ± 727 mL/min) (*p* = 0.026) (Figure 2), whereas in the anemic group the VO_2_ remained unchanged in the two conditions (ANN: 1759 ± 314 mL/min vs. ANC: 1725 ± 321 mL/min) (*p* = 0.92). Metabolic heat production (Hprod) in absolute values during exercise (Table 2) was higher in CONC (710 ± 236 W) than in CONN (645 ± 204 W) (*p* = 0.035, η_p_^2^ = 0.25), but similar between ANC (610 ± 106 W) and ANN (606 ± 107 W). Also, Hprod was not different between ANN and CONN in relative values as well (Table 2).

**Figure 3:**
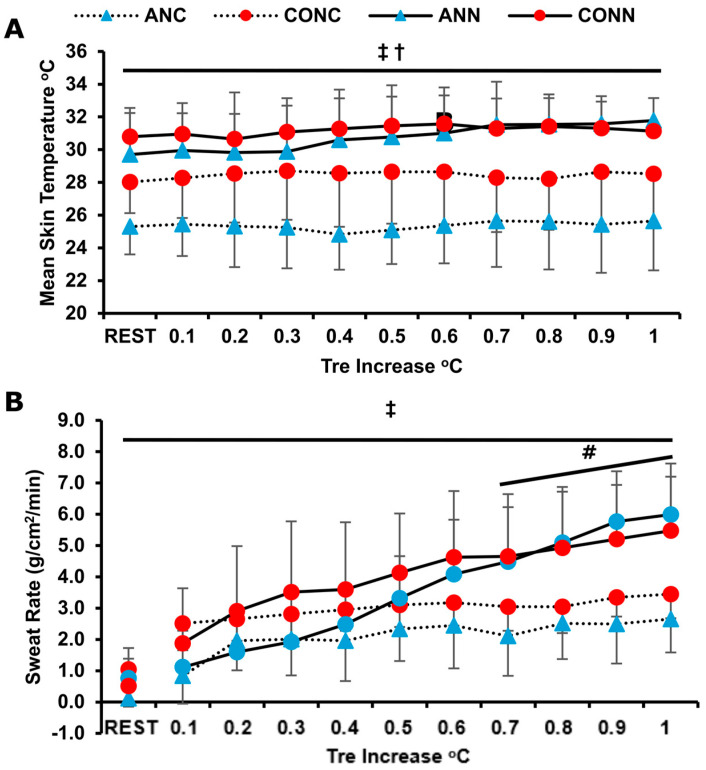
(**A**) Mean skin temperature and (**B**) sweat rate plotted against change in rectal temperature during exercise in anemic participants (n = 9) at neutral (ANN) and cold (ANC) and for controls (n = 9) in neutral (CONN) and cold (CONC) environmental conditions, respectively. ‡: Significant difference between ANC and CONC (*p* < 0.05). †: Significant difference only between ANC and ANN (*p* < 0.05). #: Significant difference between environments for both groups (*p* < 0.02). Data are presented as mean ± sd.

A group x condition interaction (*p* = 0.010, η_p_^2^ = 0.36) was found for T_skin_ accounted for by the lower T_sk_ values at rest and throughout exercise observed in ANC compared to CONC (*p* = 0.028) and ANN (*p* = 0.001) (Figure 3A). Cold exposure delayed the onset of sweating in both groups (*p* = 0.001). There was a strong tendency for difference between ANC (0.33 ± 0.2) and CONC (0.21 ± 0.1) (*p* = 0.056). The sweat rate increased as time progressed (*p* = 0.0001), and it was lower in cold environments irrespective of group (*p* = 0.004, η_p_^2^ = 0.40). Also, SwR in ANC was lower than CONC during exercise (*p* = 0.024) (Figure 3B).

**Figure 4:**
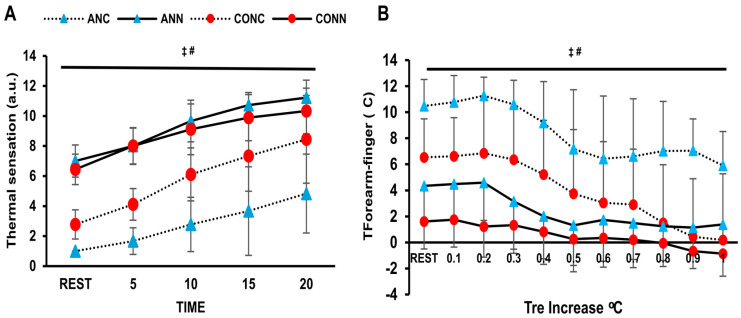
(**A**) Thermal sensation at rest and specific time points during exercise and (**B**) vasomotor activity (gradient of forearm-finger skin temperatures) plotted against change in rectal temperature during exercise in anemic participants (n = 9) at neutral (ANN) and cold (ANC) and for controls (n = 9) in neutral (CONN) and cold (CONC) environmental conditions, respectively. ‡: Significant difference between ANC and CONC (*p* < 0.01). #: Significant difference between environments for both groups (*p* < 0.05). Data are presented as mean ± sd.

There was a violation of normality for thermal sensation. Therefore, a Friedman test was conducted, indicating that there is a condition effect for both the anemic and control groups (*p* = 0.001). Thus, the nonparametric Wilcoxon was used as a post hoc test and showed a difference between cold and neutral conditions for both groups (*p* = 0.001). Lower thermal sensation values were noticed in ANC compared to CONC (*p* = 0.01) according to the Mann–Whitney test for independent groups. T_f-f_ was lower in the anemic participants than in the controls (*p* = 0.004, η_p_^2^ = 0.42) and higher in the cold than in the thermoneutral condition (*p* < 0.001, η_p_^2^ = 0.72) (Figure 4B).

## 4. Discussion

Previous studies in animals and humans with iron-deficient anemia have focused primarily on temperature regulation responses during cold exposure at rest [5,6,7]. This is the first study to examine integrated cardiovascular and thermoregulatory responses at rest and during cycling exercise in cold conditions among anemic individuals. Our main findings are that chronic mild iron-deficiency anemia affects cardiovascular and thermoregulatory responses in individuals exposed to cold air conditions at rest and during exercise. Specifically, anemic individuals showed a lower rate of rectal temperature rise and mean skin temperature, augmented peripheral vasoconstriction, and an exaggerated blood pressure response in the cold condition. Also, anemic individuals exhibited suppressed sweating rate responses. These findings suggest enhanced cardiovascular strain in anemic individuals, especially during exercise, probably inducing high cold vulnerability in this group of people.

### 4.1. Cardiovascular Responses

In young anemic individuals, elevated mean arterial pressure was recorded both at rest and during early exercise, more than in controls, in cold conditions. Although anemia is generally associated with vasodilation [38,39], this is the first study showing an exaggerated blood pressure response during cold exposure. This likely suggests increased sympathetic activation and peripheral vasoconstriction in anemic individuals. The observed accentuated cardiovascular strain could be of clinical importance and raise the risk of adverse events in cold environments, given the frequent coexistence of anemia and cardiovascular disease. In the present study, the healthy participants exhibited elevated blood pressure response at rest (~5 mmHg) and exercise (~10 mmHg) in the cold compared to the neutral condition. This difference, however, did not reach statistical significance, likely due to great individual variability [13,40]. The lower skin temperature observed in our anemic individuals in the cold is associated with increased total peripheral resistance. Based on T_f-f_, this is attributable to changes in cutaneous vasculature, although current data in young adults remain inconclusive as to whether sympathetic response also involves muscle vasculature [11,41,42]. Moreover, the hypertensive response may be driven by higher catecholamine levels in anemia, as supported by earlier animal and human research [4,16,17]. In our study, the cardiac output of anemic individuals was lower in the cold compared to the neutral condition. This was due to the lower heart rate response, commonly observed during cold exposure, especially under extreme cold conditions where body temperature is reduced [8]. Also, intriguingly, this reduced heart rate could be indicative of an attempt to attenuate cardiac output and, therefore, blunt the exaggerated arterial blood pressure response, thus altering the operating point of HR baroreflex [43]. In our study, stroke volume remained relatively stable in both groups and, thus, central venous pressure (preload) was not different among conditions and groups. This response is in alignment with previous research showing that stroke volume is typically preserved or even elevated [44,45,46,47]. As arises from above, the hypertensive response displayed by the anemic individuals is mainly attributed to enhanced peripheral resistance. However, further research is warranted in anemic individuals regarding the purpose served and the precise autonomic responses recruited in cardiovascular control under cold stress.

### 4.2. Temperature Regulation Responses

Cutaneous vasoconstriction serves as a principal defending mechanism against body temperature reduction during cold exposure [8]. In our study, the anemic group showed higher vasoconstrictor activity in cold conditions compared to controls, as assessed by the finger–forearm skin temperature gradient, which resulted in lower mean skin temperature. Despite this, anemic individuals displayed a lower rate of rectal temperature rise in cold conditions compared to controls, suggesting impaired heat-generating mechanisms.

VO_2_ response has a major role in temperature regulation in the cold [8]. In our study, the anemic participants had comparable metabolic heat production to controls in the neutral condition, but they could not increase their VO_2_ responses in the cold as the healthy participants did, and this is evident in their higher metabolic heat production both in absolute (W) and relative (W·kg^−1^ & W·m^−2^) values. Our findings align with earlier findings that iron deficiency and iron-deficiency anemia affected the metabolic responses during cold exposure in resting humans and animals. Particularly, Beard et al. [5] in their study, after controlling for body fat and menstrual cycle, reported that anemic women had lower VO_2_ values in the cold and their core temperature declined faster due to insufficient non-shivering thermogenesis mechanisms. The authors suggested that these responses are linked to lower thyroid hormone levels, since iron deficiency affects the conversion of T4 to more drastic T3, leading to diminished circulating levels of thyroxine and triiodothyronine in rats and humans [5]. This conversion to the more drastic T3 requires iron [1] and is crucial for stimulating brown adipose tissue thermogenesis, essential for energy homeostasis in cold conditions [48,49]. As thyroid hormones were not directly measured in this study, it remains to be determined whether this mechanism is responsible for not further activating the metabolism in anemic individuals during exercise in the cold.

Thermal sensation plays a critical role in initiating behavioral thermoregulation, but there is a lack of information regarding individuals with chronic iron-deficiency anemia. In our study, cold exposure reduced subjective thermal sensation in both groups, with anemic participants reporting even lower values. During exercise in the cold, their thermal sensation mirrored lower mean skin temperature, slower rectal temperature rise, and greater vasoconstriction, as indicated by the finger–forearm gradient. These findings agree with previous studies showing that thermal sensation is influenced by skin and rectal temperatures [50]. Anemic individuals’ higher cold sensitivity may reflect a compensatory behavioral mechanism for impaired heat production. On the other hand, it could be a greater intolerance, which possibly arises from their diminished inability to maintain core temperature. Crucially, from a clinical perspective, cold intolerance is connected to earlier onset of ischemia or angina during exercise in coronary artery disease patients [51,52]. This greater cold sensitivity and altered cold perception could increase dropout risk in outdoor training or occupational tasks. Further research should elucidate the thermal perception of anemic humans and the interactive effect of additional thermal factors, such as aging, hypoxia, and sleep deprivation, on thermal sensation.

To our knowledge, the present study is the first one to examine the temperature regulation responses of iron-deficient anemic humans during exercise in the cold, with previous research focusing only on resting conditions [7,17,18]. In the present study, the anemic participants also displayed lower local sweat rate in the cold in the same manner as T_sk_. In fact, skin temperature modulates sweat rate responses [53], especially during cold exposure, where a drop in T_sk_ reduces the sweat production without any observed change in body temperature [54].

The findings of this study on how iron-deficiency anemia alters cardiovascular and thermal responses during cold exposure could be applied in a wide range of areas, such as clinical practice, exercise performance, workers, and military personnel. From a clinical perspective, these findings can help identify individuals at higher risk for adverse outcomes in cold environments, particularly in populations with complex cardiovascular conditions.

### 4.3. Methodological Considerations

This study is not without limitations. During cold exposure, the sympathetic nervous system is stimulated, and, therefore, the autonomic cardiovascular responses play a pivotal role in maintaining the homeostasis of the cardiovascular system both at rest and during exercise. Given that the anemic individuals showed a reduced heart rate with a concomitant increased mean arterial pressure, it is suggested that the arterial baroreflex may be altered in this group; however, the study design did not allow for a thorough investigation of the role of autonomic regulation, especially the arterial baroreflex function. Therefore, more in-depth investigation of autonomic regulation is required in anemic populations. Moreover, the study was limited by the absence of skin blood flow measurements, since sympathetic noradrenergic vasoconstrictor nerves induce a rapid decrease in skin blood flow in cold conditions [10]. However, we attempted to address this limitation by measuring the forearm–finger skin temperature gradient, which is considered a valid qualitative index of cutaneous vasomotor tone during steady-state exercise [34]. Moreover, the small sample size and sex distribution may limit the generalizability of the results. Also, ecological validity was compromised, as the choice for light clothing does not reflect real-world attire in cold environments. In addition, a combination of cold temperatures with other environmental stressors (i.e., altitude, wet environment) could pose an additional challenge to metabolic responses [55].

## 5. Conclusions

In conclusion, our study highlights the significant impact of chronic mild iron-deficiency anemia on cardiovascular and thermoregulatory responses during cold exposure, particularly during cycling exercise. Anemic individuals displayed a slower rise in rectal temperature. They also exhibited a lower skin temperature, as well as elevated vasoconstriction and total peripheral resistance, as a compensatory mechanism. The latter responses resulted in exaggerated blood pressure, indicating exaggerated cardiovascular strain compared to controls. Given the prevalence of anemia in individuals with cardiovascular diseases and in a significant portion of the athletic population, more research is needed to adequately address the autonomic cardiovascular responses under various adverse environments. Future research should include direct measures of autonomic regulation and extend findings to larger, more diverse populations, including older adults and athletes.

## Figures and Tables

**Table 1:** Participants’ baseline hematological and anthropometric characteristics.

Characteristics	AN	CON	*p* Value
N (M/F)	9 (4/5)	9 (4/5)	
Height (cm)	169 ± 8	171 ± 8	0.62
Body mass (kg)	62 ± 8	63 ± 11	0.86
Body fat (%)	17 ± 6	16 ± 6	0.79
BSA (m^2^)	1.72 ± 0.15	1.74 ± 0.18	0.78
[Hb] (g/dL)	11.8 ± 0.4	14.1 ± 0.9	0.0001
Hct (%)	37.1 ± 1.8	42.3 ± 2.7	0.0001
Fe (μg/dL)	53 ± 16	93 ± 13	0.0001
Ferritin (ng/mL)	15 ± 9	56 ± 12	0.0001
VO_2max_ Neutral (mL·kg^−1^·min^−1^)	40.6 ± 4.7	46.3 ± 7.1	0.06
VO_2max_ Cold (mL·kg^−1^·min^−1^)	41.1 ± 4.9	46.7 ± 6.6	0.07

Data are presented as mean ± sd. BSA: Body surface area, [Hb]: hemoglobin concentration, Hct: hematocrit, Fe: serum iron.

**Table 2:** Participants’ temperature regulation parameters at rest and during exercise.

Variables	ANN	ANC	CONN	CONC
Tre Rest (°C)	37.02 ± 0.13	37.08 ± 0.15	37.05 ± 0.13	37.06 ± 0.20
Tre End exercise (°C)	38.01 ± 0.10	37.93 ± 0.15	38.02 ± 0.16	38.03 ± 0.1
Tre increase (°C·min^−1^), value × 100	2.69 ± 0.9	1.84 ± 0.9 *#	2.74 ± 0.4	2.88 ± 0.5
Exercise time to increase +1 °C or 1 h duration (min)	41.3 ± 12.1	52.8 ± 9.3 *#	35.8 ± 5.4	38.2 ± 8.8
Hprod (W)	610 ± 105	606 ± 107	645 ± 204	710 ± 236 *
Hprod (W·kg^−1^)	9.8 ± 1.4	9.7 ± 1.0	10.1 ± 1.7	11.1 ± 2.2 *
Hprod (W·m^−2^)	355 ± 48	352 ± 42	365 ± 78	402 ± 94 *
Threshold (°C) for SwR increase (g·cm^−2^·min^−1^)	0.15 ± 0.1	0.33 ± 0.2 **	0.14 ± 0.0	0.21 ± 0.1 **

Data are presented as mean ± sd. ANN: anemia + neutral; ANC: anemia + cold; CONN: control + neutral; CONC: control + cold; all groups n = 9. Tre: rectal temperature. Hprod: heat production in absolute (W), relative to body mass (W·kg^−1^), and relative to body surface area (W·m^−2^). SwR: sweat rate. *: Significant difference between environments (*p* < 0.05). **: Significant difference between environments (*p* < 0.01). #: significant difference between groups for the same environment.

## Data Availability

The data presented in this study are not publicly available due to privacy and ethical restrictions. The raw data supporting the conclusions of this article will be made available on request from the corresponding author.

## Abbreviations

The following abbreviations are used in this manuscript:

T_sk_	Mean skin temperature
T_re_	Rectal temperature
T_f-f_	Forearm–fingertip temperature gradient
VO_2_	Oxygen uptake
TPR	Total peripheral resistance
MAP	Mean arterial pressure
CO	Cardiac output
HR	Heart rate
RH	Relative humidity
SwR	Sweat rate
Hprod	Heat production
[Hb]	Hemoglobin concentration
Hct	Hematocrit
AΝΝ	Anemic group in neutral conditions
ANC	Anemic group in cold conditions
CONN	Control group in neutral conditions
CONC	Control group in cold conditions
